# Defining Blood Plasma and Serum Metabolome by GC-MS

**DOI:** 10.3390/metabo12010015

**Published:** 2021-12-24

**Authors:** Olga Kiseleva, Ilya Kurbatov, Ekaterina Ilgisonis, Ekaterina Poverennaya

**Affiliations:** Department of Computational Biochemistry and Bioinformatics, Institute of Biomedical Chemistry, 119121 Moscow, Russia; kurbah16@mail.ru (I.K.); ilgisonis.ev@gmail.com (E.I.); k.poverennaya@gmail.com (E.P.)

**Keywords:** serum, plasma, metabolomics, gas chromatography-mass spectrometry (GC-MS), multi-omics

## Abstract

Metabolomics uses advanced analytical chemistry methods to analyze metabolites in biological samples. The most intensively studied samples are blood and its liquid components: plasma and serum. Armed with advanced equipment and progressive software solutions, the scientific community has shown that small molecules’ roles in living systems are not limited to traditional “building blocks” or “just fuel” for cellular energy. As a result, the conclusions based on studying the metabolome are finding practical reflection in molecular medicine and a better understanding of fundamental biochemical processes in living systems. This review is not a detailed protocol of metabolomic analysis. However, it should support the reader with information about the achievements in the whole process of metabolic exploration of human plasma and serum using mass spectrometry combined with gas chromatography.

## 1. Introduction

Metabolites are substances with low molecular weight (<1500 Da), intermediates, and products of chemical reactions catalyzed by various enzymes in living systems. In other words, metabolites are small molecules reacting to intrinsic or environmental challenges. The metabolome, in turn, is a “snapshot” of all metabolites in the biological object at a specific time point.

Metabolomics is an essential member of the “omics” family. First, the genome and the transcriptome, made up of four bases, provide possible scenarios of the functioning of biological systems. Next, assembled from 20 amino acids, the proteome shows which protein machines are available. Finally, the metabolome indicates the reaction of the biological system to disturbances happening right now [1]. 

As the final stage of the spectrum of “omics,” metabolomics reflects the diversity of chemicals arising from the sequential and synergistic interactions of 20,000+ human genes, 60,000+ transcripts, and 1,000,000 types of proteins in the dynamically changing environment [2,3]. The information about how an organism or a cell attempts to retain nutrients while eliminating xenobiotics is vital for predicting the phenotype of a biological system [4].

Exploring the human metabolome is valuable for understanding pathophysiological processes and searching for new diagnostic and prognostic biomarkers of various disorders [5,6], accelerating drug discovery [7], and assessing the impact of diet [8,9], lifestyle [10], and other factors [11].

In the professional community of scientists working in metabolomics, the gas chromatography-mass spectrometry tandem (GC-MS) has a reputation as one of the most reliable, robust, and used analytical platforms, accompanied by a variety of spectral libraries for processing experimental data [12].

Metabolomics, as we imagine it today, appeared relatively recently. In this review, we tried to highlight some excellent methods of sample preparation, subsequent GC-MS analysis, and post-processing that have emerged over the past 20 years to provide a high-throughput characterization of small molecules in blood plasma and serum, the substrates-of-choice for many years.

## 2. Serum and Plasma Metabolome as a “Snapshot” of a Human Biochemistry

Blood and its components are the “workhorses” and favorite subjects for metabolomics analyses. For the query (blood metabolome) OR (plasma metabolome) OR (serum metabolome), the PubMed repository retrieves more than 16,000 articles published over the past 20 years. The dynamics of the increase in the number of papers is also staggering. If in 2008 fewer than a hundred studies on selected keywords were published, then in 2020, there are already more than 2000 publications.

Such attractiveness of blood for metabolomic studies is due to the low invasiveness of its extraction. Blood is usually analyzed either as serum or plasma.

The serum is a biological liquid obtained when whole blood coagulates. Coagulation is a vital process that prevents excessive blood loss from a minor wound. In a laboratory, it is common to centrifuge the coagulated blood to the bottom of the collection tube, leaving serum above the clot. The main components of serum are water, various proteins, peptides, amino acids, hormones, nitrogen compounds, various ions and salts, traces of nucleic acids, metabolites, and lipids.

Plasma is the liquid component of blood, where coagulation has been prevented. It is obtained when a clotting-prevention agent (called anticoagulant) is added to whole blood and then placed in a centrifuge to separate the cellular material from the lighter liquid layer. Common anticoagulant agents are EDTA (ethylenediaminetetraacetic acid), heparin, and citrate. After centrifugation, the remaining plasma contains fibrinogen and other coagulation factors in their original state. From the biochemical point of view, plasma is practically identical to the liquid fraction of circulating blood (except for anticoagulants). It can be said that the main difference between plasma and serum is the presence of fibrinogen and coagulation proteins. Still, there are many other more subtle differences (e.g., in eicosanoids’ levels [13,14]).

The nomadic nature of this biological fluid is also noteworthy. Blood’s metabolic profile and components provide a global “snapshot” of the transformations of substances and energy occurring in all organs and tissues. The ability of the blood metabolome to reflect internal and external perturbations [15,16] of the organism makes it one of the most important information layers for comprising a global picture of the condition of the individual at a specific point in time [17]. The versatility of the blood metabolome has provided fruitful ground for an impressive body of work investigating rapid [18,19] and slow [20,21] changes in the body. Modern metabolomic approaches allow the identification of characteristic patterns of low molecular weight compounds and associate them with the development of pathologies [22,23,24], lifestyle [25,26], and eating habits [27,28,29] in both personalized mode [10,30,31] and large cohorts [32,33].

Together, serum and plasma are the second most commonly used biofluids in metabolomic studies after urine [34] The preanalytical stages of blood processing are different for plasma and serum. Anticoagulants are added to plasma vacutainers, and coagulation enhancers are added to serum vacutainers. The question arises, are there any differences in the metabolomes of plasma and serum? If so, how do they affect the interpretation of obtained data? Studies show that none of the considered biological fluids has a clear advantage over the other in forming a representative metabolomic image of the object under investigation or the development of potential biomarkers [13,35,36]. Unless the primary plasma preparation looks more attractive: the processing is more reproducible and fast since there is no need to wait for the blood to clot, and clotting time may vary across individuals [37]. Another advantage of plasma over serum is the lower risk of hemolysis and thrombocytosis and the almost complete absence of post-centrifugal coagulation interference that can occur in serum. Plasma and serum metabolomes are comparable in “width”: the average number of unique metabolites identified in plasma and serum routinely using GC-TOF-MS equipment was 178 and 170, respectively, and 80% of this metabolic pool was present in both types of biomaterial [38]. Notably, deltas between the signal intensities of several metabolites in serum and plasma persisted. This observation means that plasma and serum offer similar analytical opportunities. Moreover, the experiments performed on plasma can be compared with those on serum and vice versa, which may facilitate the metabolic comparison of samples obtained using different protocols.

Nevertheless, the choice of the sample may significantly affect the results of experiments for several molecules of interest. Therefore features of different routinely applied protocols should be considered when comparing plasma and serum tests [39,40,41,42]. For example, the profiles of amino acids in plasma and serum differ because several amino acids are prone to conversion during clotting at room temperature (e.g., asparagine converts to aspartic acid and glutamine converts to glutamic acid) [43].

On the other hand, the serum is a more “metabolite-rich” matrix: the concentration of most metabolites in it is generally higher [42]. For example, the platelet activity marker thromboxane B2 (TXB2) concentration in plasma is 0.2–2 ng/mL, and in serum, it is already 2–178 ng/mL [44]. The volume displacement effect can partially explain this phenomenon. It is assumed that after serum deproteinization, the remaining low molecular weight components are distributed in a smaller volume, which increases their concentrations. Perhaps this was the reason for the identification of more potential biomarkers in serum than in plasma in the study of various phenotypes [45].

It is, in any case, evident that there are still more similarities between plasma and serum than differences, and it is impossible to make a clear choice in favor of one or another liquid. Fortunately, there is no need to do this since both plasma and serum are excellent matrices for metabolomic studies with broad applicability.

## 3. How Many Blood Metabolites Are There?

The potential space of organic substances that make up the metabolome is truly colossal: it lies between 10^63^ and 10^200^ unique substances [46]. Even if these theoretical spaces are reduced by several orders or even tens of orders of magnitude, they will not cease to look astronomical. Such an impressive amplification of diverse chemical information is a blessing and a curse of metabolomics at the same time [5]. At the moment, according to the HMDB 5.0, the world’s largest and most comprehensive human metabolome database [47], more than 18 thousand unique low-molecular compounds of various nature have been detected and quantified in human blood. However, the diversity of metabolites in human blood is complex and challenging to assess.

**Figure 1:**
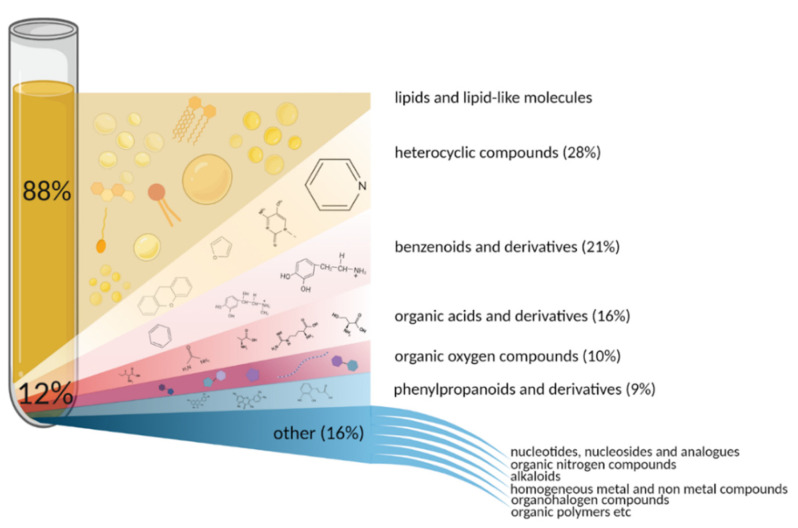
Diversity of chemical substances comprising human blood metabolome based on HMDB 5.0.

Globally, all human blood metabolites can be divided into water-soluble and lipid-soluble groups. The share of lipid-soluble molecules accounts for 88% of the metabolome (Figure 1). Lipids and lipid-like substances represent a significant and chemically diverse fraction of metabolome (>80,000 lipid molecules exist in humans, and more than 20,000 of them are found in the blood), which play essential roles in living systems. Various functional groups of lipids make them versatile machines serving as cellular barriers (various phospho- and glycolipids [48], membrane matrices (cholesterol), signaling agents (ceramide, sphingosine), and energy reservoirs (triglycerides) [49,50,51].

Non-lipid metabolites account for only about 12% of the total blood metabolome. Still, the variety of classes these substances belong to is much wider than that of the lipid-soluble fraction (Figure 1).

Metabolites differ significantly in their physical-chemical characteristics. To illustrate this diversity, we analyzed significant experimentally established and predicted properties of blood metabolites deposited into HMDB 5.0: results are presented in Supplementary Material S1.

This diversity inevitably leads to difficulties throughout metabolome research: from developing universal protocols suitable for a wide range of substances to post-processing and interpretation of obtained results. Even in panoramic mode, metabolomics cannot equally effectively explore and quantify the entire metabolome during one experiment, in contrast to more “mature” genomics, which allows sequencing the entire genome of the object under study. In the case of the proteome and metabolome, it is impossible to detect and quantify all proteins and metabolites, so researchers are forced to work under conditions of myopia, observing only part of the overall picture [52].

The concentration range of small organic molecules in blood covers nine orders of magnitude. The most abundant organic molecules are present at the level of several mM (cholesterol, urea, amino acids), and the limit of the concentrations of quantified metabolites is pM and below (various glycerophospholipids). In general, the average concentration of metabolites in the blood of healthy donors can vary within the range of ±100% (glucose, lactic acid, glutamine, glycine) due to several internal (age, sex, genetic patterns) and external (diet, circadian rhythms, and fitness) determinants [44].

## 4. Approaches of Metabolome Exploration 

Just as proteomics differs from protein chemistry in that it seeks to cover the entire spectrum of protein compounds, metabolomics differs from local analytical techniques in the breadth of view on the profile of low molecular weight molecules. Metabolites are so diverse in physical and chemical properties: a mix of volatile alcohols, hydrophilic sugars, and hydrophobic lipids, amino- and non-amino organic acids comprise the metabolome of the sample. This motivates metabolome researchers to exploit the advantages of several analytical platforms [5].

**Figure 2:**
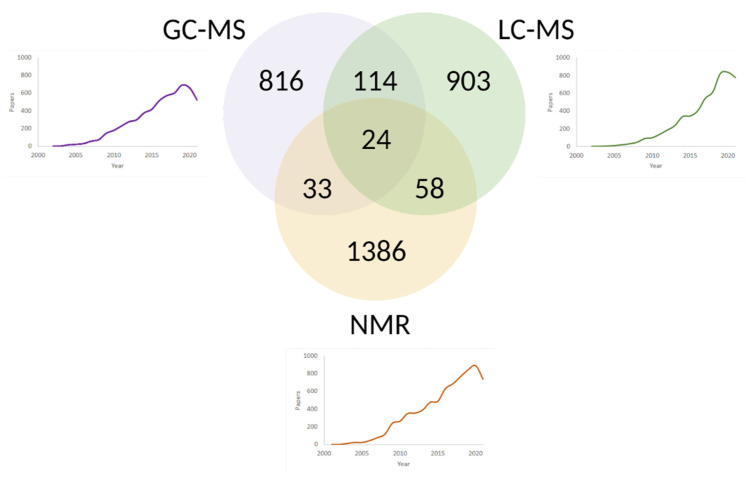
Venn diagram and timelines comprising the number of scientific publications on blood metabolome studies via LC-MS, GC-MS, and NMR up to date. The queries “LC-MS blood metabolome”, “GC-MS blood metabolome” and “NMR blood metabolome” were addressed to the PubMed repository. The leader in the number of publications is the most mature NMR technology, followed by gas and liquid chromatography in tandem with MS. Due to the different applicability of NMR, LC-MS, GC-MS for various classes of metabolites, the combination of two or even three analytical platforms has been used in 200+ metabolomics projects. The synergy is especially noticeable between GC-MS and LC-MS, used together approximately in 7% of mass spectrometry-based experiments.

High-throughput spectroscopic techniques are major tools of metabolomics. The most popular of them are nuclear magnetic resonance (NMR) spectroscopy [53] and mass spectrometry (MS) solo [54] or in tandem with gas (GC) [55,56] or liquid (LC) [57] chromatography (Figure 2). Both spectroscopic platforms provide extensive information on the composition and structure of several compounds of different chemical nature in a single analytical run.

### 4.1. NMR

The NMR method is based on the magnetic properties of the ^1^H and ^13^C nuclei. Suppose a molecule containing such nuclei is placed in a magnetic field and irradiated with a radio-frequency pulse. In that case, the atomic nuclei will go into an excited state, and the researcher will be able to register the signal of subsequent relaxation. This signal depends on the amount of the irradiated substance and ultimately contains information about the environment of the nucleus. Thus, the NMR spectrum of a substance is a superposition of signals from all resonating nuclei.

With over 20 years of extensive usage in metabolomics and over 1.5 thousand scientific papers (Figure 2), this method has proven to be a robust and reliable technique with exceptional reproducibility [58,59].

NMR requires relatively simple sample preparation [60]. However, moderate resolution and sensitivity of NMR impede the determination of low-abundant metabolites [61]. Another significant drawback of NMR is the small number of determined metabolites in complex mixtures (from 20 to 200 unique substances, depending on the resolution of NMR) in comparison with MS (potentially more than 500 identified substances). This advantage makes mass spectrometry dominant for exploring a wide range of metabolites [62].

### 4.2. Tandem of Chromatography and Mass Spectrometry

Separation-based MS techniques take advantage of both “ingredients.” Due to the characteristic pattern of the parent and daughter ions and advanced MS detectors, the specificity of the analysis is ensured without loss of sensitivity [63]. Before MS detection, the procedure of chromatography is carried out to downgrade the complexity of the sample. The sample components are moving through the stationary phase in the flow of the mobile phase. As a result, the complexity of the analyzed mixture entering the mass spectrometer decreases due to the distribution of substances between the mobile and stationary phases of the chromatographic column by their solubility, polarity, and volatility.

As the name suggests, liquid chromatography uses liquids as a mobile phase. An analyte solubility and an affinity for a sorbent in a chromatographic column are decisive physical-chemical separation parameters. Advances in LC-MS in comprehensive coverage of the metabolome are reported in more than 1000 publications (Figure 2). LC-MS is a universal platform suitable for analyzing the majority of plasma and serum metabolites.

GC-MS and LC-MS techniques can be successfully used together to provide comprehensive metabolome profiling [64]. In gas chromatography, the mixture of metabolites is carried by gas, and the compounds are divided in the column space by their volatility. GC-MS has long been used for metabolome profiling due to its separation capacity, sensitivity, and selectivity [12]. Reproducible molecular fragmentation patterns of GC-MS make it one of the most reliable tools for exploring metabolomes. However, the application of GC-MS is limited to volatile compounds, and a large portion of small molecular metabolites are within the range of GC separation.

Due to its excellent ability to separate complex chemical mixtures, two-dimensional gas chromatography is gaining popularity in metabolomics [65]. Series connection of two chromatography columns with different polarities allows separating compounds that co-elute from the first column. This technique becoming the new frontier of GC-MS-based metabolomics is used to characterize several classes of chemical compounds for panoramic and targeted studies of various biological samples, including blood plasma and serum (see Section 5.2.5 “Multidimensional chromatography” for further discussion).

Chemical derivatization before GC significantly improves the volatility and thermal stability of polar and non-volatile metabolites. Therefore, the content of chemical compounds that GC-MS can analyze is expanding [66]. We will build further narratives based on GC-MS technology, which offers a compelling balance of sensitivity (more sensitive than NMR) and reliability (more robust than LC-MS).

The monumental research by Psychogios and co-authors highlighted metabolites routinely found in liquid blood components [44]. The study showed that out of the total pool of 4229 metabolites (including lipids) known at that time, NMR measured 1.2%, GC-MS 2.1%, MS/MS with electrospray ionization (lipids profiling) measured 2.3%, direct flow injection -mass spectrometry (lipids profiling) measured of 79.9%, and tandem MS with direct flow injection was able to access 3.3% of the total serum metabolome.

Each technology used in metabolomics has a unique set of strengths and limitations (Table 1). It is challenging to prudently select an appropriate metabolomics platform equally well suited for studying the entire wide range of metabolites circulating in plasma and serum. However, optimal solutions can be determined for individual classes of substances. For example, the vast majority of identifications in lipid-rich serum and plasma highlights the limitations of GC-MS, which provides less advantageous lipid profiling than LC-MS [67].

## 5. Workflow of GC-MS Analysis of Blood Metabolome

### 5.1. Sample Preparation

**Figure 3:**
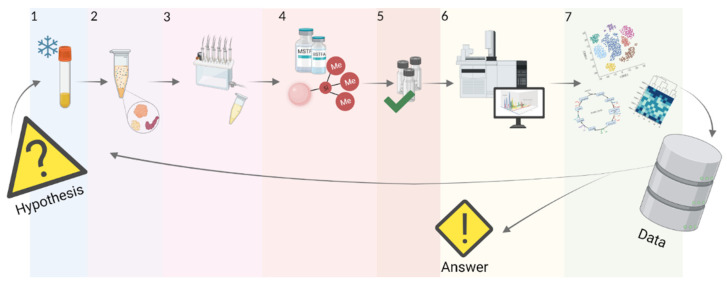
Stages of standard GC-MS analysis of plasma or serum samples. In a hypothesis-driven study, a metabolomic experiment starts with formulating a hypothesis, which further work will be aimed at confirming or refuting. Preanalytic operations involve quenching enzymatic processes (stage 1) in every biological sample from a representative sample. Further, the sample is purified (stage 2) from interfering protein molecules, followed by liquid or solid-phase extraction (stage 3), which allows the release of metabolites from the plasma or serum matrix and concentrates them in a smaller volume. Next, extracted metabolites are derivatized (stage 4) to improve their volatility and thermal stability. After ensuring that the quality criteria (stage 5) are met, the researcher performs a gas chromatography-mass spectrometric experiment (stage 6). Finally, data processing (stage 7) provides the researcher with either an answer to the original question or the basis for starting a new data-driven study [68].

The efficiency of metabolomic analysis largely depends on the stage of sample preparation. Aberrations at this stage affect the list of detected and identified molecules, the quality of the data, and, as a result, the biomedical interpretation of obtained results. Therefore, the choice of sample preparation method mainly depends on the type and volume of the sample, the physical-chemical properties of the analytes being measured, and the analytical platform used for the analysis. Metabolic analysis of plasma or serum by GC-MS involves several sequential sample preparation steps, including quenching, extraction, and derivatization. We have described each of these stages below (Figure 3).

#### 5.1.1. Quenching

Biological samples can be divided into metabolically active, whose metabolic composition can change over time (cells, tissues), and metabolically inactive, whose metabolic profile is “fixed” (saliva, urine). For metabolically inactive samples, minimal sample preparation is usually applied. Highly active samples require metabolic processes to be quenched [69,70]. Plasma and serum are biological samples of moderate metabolic activity, occupying an intermediate position between inactive urine and active cells, so they still may need a quenching stage (Figure 3, stage 1) [68].

To quench metabolic processes in plasma or serum, it is necessary to abruptly stop enzymatic activity by changing the medium’s polarity, temperature, or pH. Generally, quenching of enzymatic processes in plasma and serum coincides with the extraction of metabolites with an organic solvent.

#### 5.1.2. Protein Cleanup

Interfering proteins presented in plasma and serum may suppress analytical signals of metabolites. Therefore protein molecules should be removed in advance to reduce the complexity of the sample under study and improve the peak resolution of metabolomic profiling. The precipitation procedure is often used to purify plasma or serum from protein contaminants. Precipitation is accomplished by changing the pH and polarity of the solution. As a result, intramolecular interactions are disrupted, the protein denatures, aggregates, and falls out of solution [71,72]. For plasma and serum, the excess amounts of organic solvents such as methanol or acetonitrile [72,73] are used, followed by high-speed centrifugation and separation of the supernatant (Figure 3, stage 2).

It is noteworthy that plasma and serum represent a hydrophilic environment with limited solubility of hydrophobic metabolites such as lipids, fatty acids, steroids, and thyroid hormones. Efficient transport and distribution of these hydrophobic compounds in blood plasma in vivo are achieved due to their interaction with proteins. During the precipitation of proteins, a certain part of the metabolites is lost. To minimize this effect, there is a technique that allows the extraction of coprecipitated metabolites due to the enzymatic cleavage of precipitated proteins; however, the reproducibility of this method requires additional evaluation [74]. The combination of adding an excess of a strong organic solvent such as methyl tert-butyl ether (MTBE) with a subsequent extraction step also increases the coverage of the metabolome [75].

#### 5.1.3. Extraction

Extraction releases metabolites from the biological matrix and concentrates them in a smaller volume (Figure 3, stage 3). The breadth of the research problem dictates the choice of extraction methods.

Target metabolomics allows to qualitatively and quantitatively characterize a specific, often relatively narrow, group of metabolites [76]. In this case, at the extraction stage, it is required to reduce interference from off-target compounds and increase the completeness of extraction of target groups of metabolites.

Panoramic metabolomics is aimed at the broadest possible coverage of a wide variety of chemically diverse metabolites [76]. For such global tasks, an unselective extractant is selected that will effectively extract substances belonging to different chemical classes to ensure adequate depth of metabolite coverage and represent the actual composition of the sample under study. For metabolomic studies of serum and plasma, liquid-phase and solid-phase extraction are used.

Liquid extraction of metabolites can be performed by one- or two-phases systems. Single-phase extraction systems are of particular interest since they decrease the complexity of the experimental procedure and allow for the simultaneous deproteinization and extraction of a comprehensive metabolic fraction. In addition, such designs are attractive for studies on a limited amount of available biological material, inadequate for multiple specific protocol protocols suited for different compounds.

Biphasic extraction is based on the metabolites transfer from one liquid phase to another immiscible liquid phase in which they are more soluble. The most frequently applied organic extractants include acetonitrile, chloroform, acetone, methanol, ethanol, and mixtures at various ratios. The solvents and their proportions can significantly affect the total number of metabolites extracted.

An organized attempt was made to compare various extraction options with each other and select those conditions under which it is possible to extract the largest number of metabolites from the plasma with maximum efficiency: the choice of the extracting agent, its volume, as well as the time and temperature at which the extraction took place, was evaluated [72]. The temperature of the solvent does not affect the peak area at all or shows a slight increase in the case of acetone. A much more critical parameter turned out to be the volume and content of the solvent. Nine parts of methanol:water mixture (8:1, *v/v* [72]), added to one part of a plasma, provide optimal results in terms of completeness, efficiency, and reproducibility of extraction in comparison to other tested solvents (ethanol, acetonitrile, acetone, chloroform). The stability of the procedure for the extraction of low molecular weight compounds from plasma with methanol is also emphasized in a similar scientific work [77]. To increase the range of extractable metabolites (for example, for the reliable recovery of fatty acids), many protocols use complex solvent combinations, such as methanol:chloroform or methanol:chloroform:water [72]. For example, the protocol by O.Fiehn uses acetonitrile:isopropanol:water (3:3:2, *v*/*v*) mixture for extraction. The combination of hydrophilic, lipophilic, and medium-polarity solvents demonstrated high analytical precision and comprehensiveness of extracted metabolome [56].

Another solvent mixture of methanol:chloroform (3:1, *v*/*v*) mixture, which, due to nonpolar chloroform, provided better extraction of lipophilic metabolites from the serum sample than methanol alone [78]. The MeOH:MTBE:H_2_O mixture (2:10:3, *v*/*v*/*v*) was also highly appreciated for its versatility for various chemical classes in comparison with pure methanol and a mixture of methanol:water (3:1, *v*/*v*) with subsequent treatment with a mixture of chloroform:water (3:1, *v*/*v*) [77].

The composition of the extraction mixture and the extraction time are not the only parameters that can be varied to achieve better recovery. Ultrasonic stimulation can also increase the degree of extraction. It was shown that after four minutes of ultrasonic extraction (40 kHz, 350 W), the intensities of most of the 570 resolved peaks increased compared to two and 10 min of the traditional vortexing [79]. Moreover, by changing the pH, one can also influence the type and number of extracted metabolites. An experiment with blood components showed that the sum of unique molecular features on the chromatography-mass spectrum after extraction at pH 2, pH 7, and pH 9 is 45% more than extraction in a neutral medium [80].

Solid-phase microextraction (SPME) is based on redistributing substances between phases due to sorption or ion-exchange processes. SPME is gaining popularity as a method for sample preparation in metabolomics experiments providing significant reduction in matrix effects [81,82]. SPME process can be fully automated, allowing for high sample throughput and improving method repeatability [83,84]. Among the advantages of SPME, the diversity of commercially available extraction phases manufactured in standardized conditions is noteworthy [85]. For the extraction of a broad metabolome, relatively versatile silica-based C18 resins [86] and complex multicomponent and multilayer coatings, e.g., DVB/CAR/PDMS (divinylbenzene/carboxene/polydimethylsiloxane) are used [87]. Such coatings make it possible to adapt the average pore size in different sorbent layers to different sizes of extracted analytes.

The polyacrylate (PA)-coated SPME cartridge has shown excellent results for recovering volatile organic metabolites from liquid biological samples. When used, the number of well-resolved peaks and their total intensity is higher than with PDMS/DVB or CAR/PDMS coated cartridges [88]. SPME with DVB/CAR/PDMS sorbent mixture made it possible to extract and subsequently identify almost 300 unique metabolites, including hydrocarbons, amines, ethers and esters, alcohols, carboxylic acids, thiols, terpenoids, and heterocyclic compounds, etc. [89].

Moreover, when comparing various complex coatings for solid-phase extraction (PDMS/DVB, PA, DVB/CAR/PDMS, CAR/PDMS, PDMS, and PEG (carbowax-polyethylene glycol)) of volatile organic metabolites, it is also the CAR-DVB-PDMS phase which allowed to detect the largest number of metabolites (63% of the pool of identifications for the sum of all methods) [90].

However, absorbents are rarely used for panoramic profiling [89], but they show good results for extracting certain classes of substances. Thus, polar sorbents (carbowax–divinylbenzene) are effective for extracting polar (alcohols, ketones, nitroaromatics) analytes, and nonpolar sorbents (PDMS) are effective for extracting nonpolar analytes (polycyclic aromatic hydrocarbons, benzene/toluene/ethylbenzene/xylenes) [91]. Solid-phase extraction revealed statistically significant differences between metabolomes of patients with breast cancer and healthy people for several volatile organic compounds (including benzene derivatives, terpenoids, ketones, sulfur compounds, and phenols) [92,93].

#### 5.1.4. Derivatization

Derivatization can be applied before or after chromatographic separation (Figure 3, stage 4). In GC, pre-column derivatization is much more common. Post-column derivatization is quite an exotic procedure enhancing the detectability of the analytes through rapid physical-chemical conversion (pyrolysis, catalytic hydrogenation, etc.). In this case, the chromatogram of the initial mixture is recorded, and then the substance is modified for more accurate mass spectrometric analysis. For example, for post-column derivatization, a dehydrogenation microreactor installed between the GC and MS was used. Since only six-membered rings without quaternary carbon atoms undergo aromatization, this post-column dehydration makes it possible to differentiate between cyclopentane and cyclohexane hydrocarbons [94]. As part of this review, we will focus on predominant pre-column methods.

During GC-MS analysis, volatile low molecular weight compounds pass through a chromatographic column heated to about 300 °C and higher. However, most metabolites (e.g., glucose, lactate, pyruvate, palmitate, etc.) have higher boiling points due to their polar functional groups. They, therefore, are not volatile at the highest temperature allowed for a GC system.

Pre-column chemical derivatization protects polar functional groups, improves the volatility and thermal stability of the molecule [95]. The replacement of acidic protons of amino-, hydroxyl-, carboxyl-, and thiol-groups with other groups (silyl-, alkyl formate-, etc.) weakens intermolecular interactions, decreasing the boiling point and polarity of the metabolite, and increasing its stability [96,97].

The choice of the optimal derivatization reaction for GC-MS-based metabolomics is not always a simple task because there are a plethora of various compromises, more or less suitable for specific molecular targets. Most popular and universal derivatization protocols use trimethylsilylaton strategy and its variants [55]. Alternative variants (alkylation and acylation complete the list of the top three most abundant techniques) expand the “toolbox” of derivatization and will also be described below.

**Figure 4:**
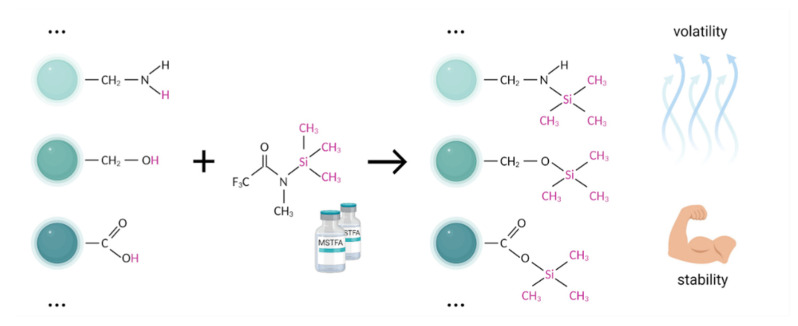
The typical scheme of trimethylsilylation. Bimolecular nucleophilic substitution (SN2) replaces the active hydrogen (-OH, -COOH, -NH, -NH2, and -SH) by the trimethylsilyl group. Compared to the initial compounds, silylated derivatives generally are more volatile and thermally stable, thus yielding advanced separation of symmetric and narrow chromatographic peaks.

Silylating agents replace the active proton in many functional groups, including OH, COOH, SH, NH, CONH, POH, and SOH with trimethylsilyl (TMS) group [97]. The good volatility and stability characteristics make silylated derivatives highly suitable for GC-MS analysis (Figure 4).

The most commonly used silylation agents are N,O-bis-trimethylsilyltrifluoroacetamide (BSTFA), N-methyl-trimethylsilyltrifluoroacetamide (MSTFA), and N-tert-butyldimethylsilyl-N-methyltrifluoroacetamide (MTBSTFA). Among popular trimethylsilylacetamides, MSTFA is the most volatile, mild, and versatile reagent for the complex profiling of metabolites of blood components [56]. The silylation potential of BSTFA is close to that of MSTFA, but the volatility is slightly lower, which may positively affect the ease of use of this derivatizing agent. Moreover, the stability of BSTFA derivatives ensures low noise and moderate detector fouling [97] (Figure 4).

The molecular weight of the initial molecule plays an essential role in the derivatization. If the molecule has a low molecular weight, there are no fundamental differences between the TMS agents. For relatively large metabolites (dicarboxylic acids, styrenes, monohydroxy-polycyclic aromatic hydrocarbons) without hindered functional groups (9-hydroxyfluorene, sugars), MTBSTFA is an excellent solution. A significant advantage in using MTBSTFA is the absence of highly volatile by-products that interfere with early eluting peaks [98].

Direct silylation of keto- and aldehyde groups produces several redundant compounds, complicating chromatograms’ qualitative and quantitative interpretation [99,100]. To avoid chromatographic artifacts (e.g., keto-enol tautomerization), oximation by hydroxylamines or alkoxyamines is used before silylation. It protects keto- and aldehyde-groups, preventing cyclization of sugars and decarboxylation of α-keto acids [101]. The methoximation takes 1–2 h when heated (usually up to 37–70 °C) or overnight at room temperature [72,102].

It is vital to carry out silylation in an anhydrous medium, such as pyridine, since active hydrogens of water molecules react vigorously with silylating agents. Anhydrous pyridine acts as an acid scavenger and accelerates derivatization [103]. Therefore, the silylation is usually completed at room temperature or heating within an hour to 60–70 °C [104]. Controlled microwave radiation reduces reaction times to minutes [103,105].

An important disadvantage against the background of all the listed advantages of TMS-derivatization is the deteriorative effect of the excess of the TMS-reagent on the sorbent of some types of chromatographic columns (e.g., carbowax (polyethylene glycol) columns) [106].

Alkylation is an alternative derivatization strategy that can be used in metabolomic profiling. Ideologically, alkylation (or arylation in the case of the reagent with aryl group) is similar to silylation, based on nucleophilic substitution of the active hydrogens from the -OH, -COOH, -SH, -NH, or -CONH groups with an aliphatic or aliphatic-aromatic group. The alkylation products are less polar and more stable than the initial molecules, which, if necessary, makes it possible to isolate and “preserve” derivatives [97,107].

Popular alkylating agents are short-chain alkyl- or aryl-halides, in which the cationic moiety carries a specific property, and the anionic moiety is responsible for specific reactivity. In addition to them, dialkyl acetals, diazolalkalanes, pentafluorobenchyl bromide (PFBBr), boron trifluoride in methanol or butanol, tetrabutylammonium hydroxide, dimethyl sulfate are used.

PFBBr alkylates phenols, thiols, and carboxylic acids. Alkyl bromides are used mainly for the derivatization of carboxylic acids and tetrabutylammonium hydroxide (TMH) for low molecular weight amines and carboxylic acids [97].

Alkylation has several features, both attractive to the researcher and undesirable. The advantage of derivatization through alkylation is a wide range of reaction conditions, varying from strongly acidic to strongly basic. Another valuable property is reaction speed. For example, dialkyl acetals react so quickly with functional groups of carboxylic acids, phenols, and thiols that this reaction can be used in flash derivatization directly at the injection port. On the other hand, some alkylating reagents of this type (diazomethane and dimethyl sulfate) are extremely toxic, and some (boron trifluoride) are very unstable even at low temperatures and are extremely sensitive to moisture. Such nuances significantly complicate the process of alkyl derivatization [97].

Like the derivatization approaches described above, the nucleophilic substitution of the active hydrogen of the polar group with the RCO group of the acylating agent allows reducing the polarity of small molecules and improve their behavior in the chromatographic column, although not so effective as silylation or alkylation. Acylylation gives the ability to derivatize a wide variety of compounds for GC analysis, especially amines, amino- and organic acids in serum and plasma [108,109]. For example, a comparison of the ethyl chloroformate derivative of low molecular weight compounds in blood serum made it possible to reliably distinguish between clusters of healthy volunteers and patients with uremia [110].

Unlike the more popular silylation, acylation can be performed in an aqueous medium, which is convenient for metabolomic profiling of plasma and serum [106]. In addition, rapid acylation [111] in an aqueous medium allows some highly volatile polar metabolites, for example, alcohols or phenols [107] to be derivatized without fear of their premature evaporation (which, for example, can happen during aggressive drying before derivatization with silylating agents). Another important advantage of acylation is its amenability to a wide range of chromatographic and mass spectrometric systems due to the simple separation of the reaction products from the reagents [106].

The most popular acylating reagents are chloroformates with the simplest alkyls (methyl, ethyl, isobutyl), which are well suited for analyzing amines, phenols, and carboxylic acids. The classic version of the reaction proceeds in pyridine and requires the corresponding alkyl alcohol (methanol, ethanol, isobutanol). Identical radicals of alcohol and chloroformate are needed to eliminate the probability of the formation of different derivatives for the same acid. After adding an excess of chloroformate, the formed derivatives are extracted, for example, with chloroform [111,112].

The main disadvantage of derivatization with alkylformates is the smaller coverage of metabolome: for example, the protocol is generally not suitable for carbonyls and amides due to their precipitation. Another critical disadvantage, especially compared with silylation, is the limited spectral databases of acylated molecules, making them challenging to identify [55,106,108].

Metabolomics methods employing derivatization are more complex than direct analysis without chemical modifications since the experimenter requires additional intervention. However, in most cases, the advantages of derivatization outweigh its disadvantages, and for this reason, it is still widely used in analytical practice [100]. Moreover, with the democratization of autosamplers that allow chemical derivatization immediately before analysis [12], metabolomic profiling becomes less laborious, and the results obtained become more reliable and reproducible.

### 5.2. Gas Chromatography

Gas chromatography allows the separation of a vaporized mixture of substances due to differences in the speed of movement of individual components in the flow of the gaseous mobile phase along with the stationary phase of the thermally controlled column.

#### 5.2.1. Injection

**Figure 5:**
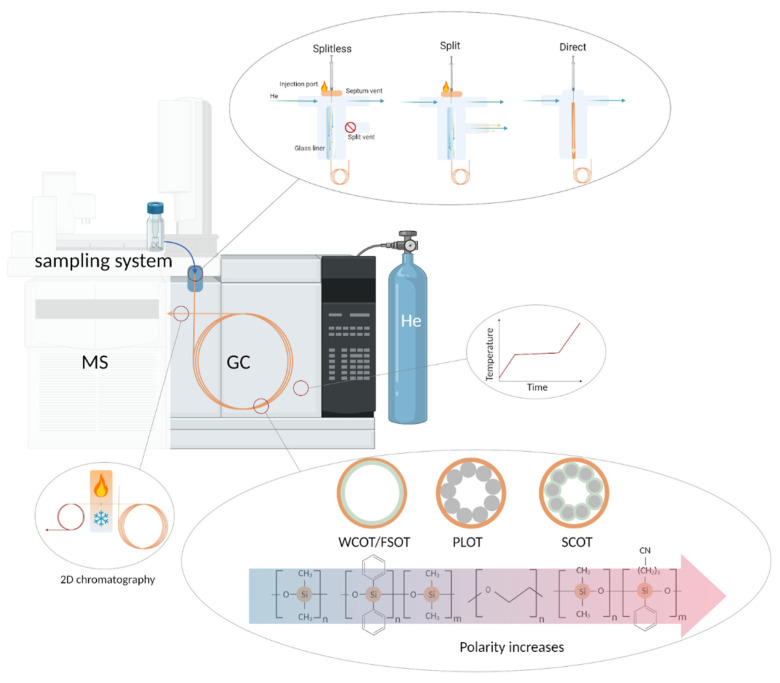
Schematic illustration of major units in the GC system. Abbreviations in the figure: MS—mass spectrometry, GC—gas chromatography, 2D—two-dimensional chromatography, WCOT-wall-coated open tubular column, PLOT-porous-layer open tubular column, SCOT-support-coated open tubular column, FSOT-and fused silica open tubular column.

Several microliters of the sample are uploaded into the GC system through an injection port (Figure 5). The port is generally heated to a high temperature sufficient for instantaneous sample evaporation but not exceeding its decomposition temperature. Depending on the purpose of the experiment and the concentrations of the target components, injection of the sample can be effected in three modes: direct, split, and splitless. As expected, each mode has its area of applicability, advantages, and drawbacks. Thus, the direct mode is used for thermally labile compounds to avoid contacting the hot injection port and direct the sample directly to the column. In splitless mode, the entire sample is fed to the chromatographic column, but it is vaporized at the injection port. This approach allows the analysis of low-copy compounds. In split mode, only a part of the total sample volume after evaporation enters the column. Dilution of the highly concentrated analyte with gas is intended to make the peaks well resolved and prevent overloading of the column. As expected, at a high split rate (1:400, when 400 parts of the carrier gas dilute one part of the vaporized sample), a low amount of sample injected into the column leads to the low sensitivity of the method. On the other hand, when the split ratio is low (for example, 1:30), the introduction rate into the column increases as the sensitivity increases.

The prepared sample enters the chromatographic system through the injection port, which is heated to a high temperature sufficient for instant vaporization of the sample. The majority of GC systems perform two types of sample introduction: split (when the sample is “diluted” by a highly pure carrier gas) and splitless (when most of the sample is uploaded into the column). Alternatively, injection directly into the chromatographic column can be applied to thermally unstable samples and trace analytes. The column itself is a long silica tube filled from the inside with various polymeric substances of different polarity (due to different functional groups in polysiloxanes) acting as a solid or liquid stationary phase. The column is placed in an oven, the temperature in which is maintained constant (isothermal mode) or changes during the wound (temperature-programmed mode). Two capillary columns with different characteristics can be connected in series through a modulator for advanced separation. After separation, eluents get into the detector.

Highly pure H_2_, N_2_, and He are used as a mobile phase, carrying a gaseous sample through the column with a minimal effect on the process of chromatographic separation and subsequent mass spectrometric detection. The required level of chromatographic separation primarily determines the choice of carrier gas. Among the three popular gases, 99.9999% He has a priority for complex plasma and serum matrices; N_2_ is also widely used due to its low operational cost. H_2_ is less popular because it is flammable and not entirely inert: it can react with mixture components at high temperatures.

#### 5.2.2. Thermal Conditions

After injection, the sample, in whole or in part, enters the chromatographic column, which is in the oven operated in either isothermal or temperature-programmed modes. In isothermal mode, a constant column temperature is maintained during the entire run. This solution is optimal for separating relatively simple mixtures, the components of which have similar RTs. Naturally, for the analysis of a complex sample, the components of which have very different boiling points, it is problematic to choose one temperature that will be maintained throughout the run and provide good separation in an adequate time. Temperature programmed gas chromatography (TPGC) involves heating the column at a controlled rate during the run and then returning to the starting point. Temperature increases speed up the movement of analytes through the column, yielding decreased RTs and duration of analysis (Figure 5).

The temperature gradient allows lower detection limits and improved peak shapes (especially late eluting peaks). The disadvantage of TPGC is a higher noise level at high temperatures compared to isotherm and the need to cool the column between analyses [113].

Interestingly, the isothermal analysis may provide higher internal efficiency than temperature-programmed analysis when analyzing the same complex mixture. However, the higher separation power of isothermal GC is achieved at the expense of extremely long run times, which ultimately last about 1000 times longer than TPGC runs [114]. With the same analysis time, the temperature-programmed option allows 2–3 times more peaks to be resolved [115]. In this regard, various temperature gradients are used to reduce the analysis time and competently resolve the signals [116].

Moreover, separation efficiency can be increased using thermal gradient gas chromatography (TGGC). The essence of this dynamic method is that each section of the column heats up independently, achieving a focusing effect, improving the shape of the peaks, and reducing the noise level. According to theoretical models on normal alkanes, peak performance and resolution in a 100 cm open tubular column operating in TGGC mode are 10–13% higher than in TPGC [117,118]. The need to accurately and rapidly produce and vary thermal gradients along the column significantly complicates the widespread adoption of this method. However, new technologies implying the simultaneous use of resistive heating and convective cooling leave hope for its popularization [118,119].

#### 5.2.3. Solid and Liquid Stationary Phases

The chromatographic column can be packed or capillary. A packed column is a metal or glass tube up to 5 m long and about 3 mm in diameter, filled with a finely powdered stationary phase. Packed columns are rarely used today because the separation efficiency they can provide is hundreds of times less than the efficiency of more advanced capillary columns [120], prepared from high-purity silica. Capillary columns are generally much longer (5–150 m) and thinner (0.05–0.53 mm). This geometry provides a high-speed movement of the sample along the column, allows one to work with microvolumes, and obtain chromatograms with better resolution. In modern capillary columns, stationary phases are applied only on the walls in four ways (Figure 5): wall-coated open tubular (WCOT), porous-layer open tubular (PLOT), support-coated open tubular (SCOT), and fused silica open tubular (FSOT). In the WCOT variant, column walls are coated with a liquid polymer, acting as a stationary phase [121]. In the PLOT column, conglomerates of porous particles are deposited in the inner wall. The inner walls of SCOT columns are lined with a layer of supporting material onto which the stationary phase is attached. FSOT column is a modification of WCOT, in which the capillary is made of fused silica, making it stronger, more flexible, and more inert than the WCOT predecessors. The introduction of crosslinking technology allowed the use of stable thick films in WCOT columns and their modifications, making the SCOT type columns practically irrelevant.

In addition to the column geometry and the method of applying the stationary phase, the stationary phase’s composition also affects the analyte’s behavior in the chromatographic system. Therefore, classical polyethylene glycols and polysiloxanes of various compositions, polarities, and thicknesses (Figure 5) are used. Moreover, ionic liquid SPs exhibit a “dual-nature”, allowing the separation of polar and nonpolar compounds and extending the temperature range at which the column can be operated [122].

A variety of stationary phases are used for blood analysis [12]: e.g., 95% dimethyl/5% diphenyl polysiloxane is popular low-polarity phase [123], and 50% phenyl/50% dimethylpolysiloxane often used as middle-polarity one [124]. When targeting specific metabolites, the phases are selected based on the target compounds. For example, in the analysis of fatty acids in plasma, polyethylene glycol was used [125].

#### 5.2.4. Retention Times and Indices

Depending on the column’s stationary phase, various intermolecular interactions (Van der Waals and dipole-dipole forces, hydrogen bonding, etc.) and polarity of individual compounds of the mixture will determine the strength of its retaining in the column. The stronger interaction between column and compound, the longer its retention time (RT). Compounds with lower boiling points and polarity tend to rapidly transfer through the column and have shorter RTs [126]. However, RT depends not only on the physical-chemical properties of the certain compound but also on various technological aspects of the CG method applied (e.g., column characteristics, thermal conditions, etc.). One of the concepts allowing identification and increasing the convergence of interlaboratory results consists in fixing all possible parameters of the system (the column used, the nature and flow of the carrier gas, temperature conditions) so that the absolute RTs carry information at a qualitative level. Nevertheless, it is difficult to imagine standardization of this level in the entire metabolomics community.

An alternative well-established practice suggests using retention indices (RI) for standardized comparison between different analytical parameters and different GC systems used in different laboratories.

The Kovats index is a pioneer among retention indexes built on a series of n-alkanes (C7–C40, C8–C20, C21–C40) [127]. In addition to n-alkanes, the series of fatty acid methyl esters (FAME) or the so-called M-series, which consists of alkyl-bis-(trifluoromethyl)-phosphine sulfides (CF_3_)_2_P(S)C_n_H_2n+1_ are used [128]. A homologous batch is added to each sample. Each homolog has a specific point on the retention index scale, and all other compounds are assigned a specific index based on the “coordinates” of the standards. Thus, unlike absolute RT, the compound index between the two reference compounds will remain constant even if the RTs of these analytes change.

Using RI as the only parameter for identifying a compound is risky because RI is not a unique characteristic inherent in only one and no other chemical substance. However, RI is excellent for orthogonal confirmation of identification, supported by mass spectrometry results [129].

#### 5.2.5. Multidimensional Chromatography

In some cases, even high-resolution gas chromatography may be insufficient for the reliable separation of complex biological mixtures.

With two- or even three-dimensional [130] chromatographic systems, that run the sample through two orthogonal columns in series, advanced resolution can be achieved. The second column is usually much shorter than the first, is filled with a different stationary phase, and operates at a higher temperature. If two metabolites leave the first column simultaneously, then the second column can provide separation due to a different stationary phase and temperature conditions [123,131,132]. For example, 100 metabolites in blood were identified using this method, divided in the first column by volatility and in the second by polarity [133].

In another study, which compared serum analysis by one-dimensional and two-dimensional gas chromatography in combination with mass spectrometry, there was a threefold increase in both the total number of peaks (490 ± 26 and 1571 ± 174, respectively) and the number of identified metabolites (348 ± 16 and 1099 ± 118, respectively) in the case of GC×GC-MS [134].

A modulator is located between the columns in a 2D chromatographic system, regulating the temperature or pressure [135]. The modulator continuously accumulates small fractions of the substance from the first column and sends the already concentrated portions to the second column with short pulses for additional separation.

Focusing gives an additional advantage: high and narrow chromatographic peaks (about 100 ms at baseline [65]) increase the resolution and sensitivity of the analysis [135,136,137].

### 5.3. Mass Spectrometry

After chromatographic separation, the analytes eluting from the column pass through a heated transfer line and interact with an MS detector. This interaction generates a response, which could be digitized and transferred to the data system. The magnitude of the signal from a certain molecular ion (or its fragments) and time from the moment of injection are used to generate a chromatogram.

#### 5.3.1. Ionization

Mass spectrometers separate ions according to their mass-to-charge ratios (*m*/*z*). The fundamental laws of nature dictate the need to work with ions: the electromagnetic field providing *m*/*z* separation can interact only with charged particles. Thus, the first step required for the mass spectrometric detection of a substance is the conversion of molecules to ions.

Electron impact (EI) is the most common ionization method in GC-MS metabolomics. As the name suggests, ionization occurs due to the collision of electrons with gaseous molecules of a substance. The hot filament emits the electrons, which are accelerated by the high voltage towards the ionization chamber. Typically, the electron energy is 70 eV since, at this energy, it is possible to obtain stable mass spectra with a high degree of ionization and fragmentation of the molecule.

When bombarded with such a beam of energetic electrons, the gaseous substance loses its electron and becomes a molecular ion M^+^. EI is a hard ionization method, and in most cases, due to significant fluctuations in the electric field around the neutral molecule, the original molecular ion is shattered into fragments.

It is important to note that this fragmentation is reproducible between different experiments and instrumental solutions. High stability, reproducibility, and specificity of fragmentation spectra have allowed the creation of extensive repositories (FiehnLib GC-MS Library [129], Golm Metabolome Database [138], Mass Bank [139], National Institute of Standards and Technology (NIST) [140], METLIN [141], and several other sources) and re-use the deposited data for spectral identification.

In addition to electron ionization, chemical ionization (CI) can be used in GC-MS metabolomics. In CI, new ionized particles are formed when a gaseous molecule interacts not with electrons, as in EI, but with intermediate reactive ions bombarded by electrons. Chemical ionization can be divided into two subtypes, positive (PCI) and negative (NCI). During PCI, the reagent gas (methane, isobutane, ammonia) has a lower affinity for the proton than the analyzed molecules, and the proton is transferred from the reagent gas ions to the analyte molecules forming positively charged ions. In the classic NCI version, the reagent gas contains water due to ionization of which OH-anions are formed.

The GC-CI-MS platform made it possible to create a reproducible and sensitive method (up to 10 pg/mL) for the determination of trace amounts of testosterone and nandrolone esters in blood plasma, showing the linearity of the quantitation in the range of 100–2000 pg/mL [142]. PCI was used for the quantitative analysis of dimethylamine [143]. NCI-MS is also used for analyzing endogenous metabolites in human serum [144], for example, for the study of the PPB-derived eicosanoids in human serum [145].

During electron capture negative chemical ionization (ECNCI), the buffer gas slows down the electrons emitted by the heated filament, lowering their energy to ~2 eV. This option is well suited for molecules with high electron affinity (e.g., metabolites modified by pentafluoropropionyl [146]). Decelerated electrons are more easily captured by molecules, resulting in the formation of negatively charged ions.

Chemical ionization is considered a mild technique because it leads to less fragmentation and a greater chance of encountering an intact parent ion [147]. However, in metabolomics, CI is used less frequently than EI [148]. The lesser popularity may be explained because the metabolomic profile obtained using chemical ionization usually contains fewer identified compounds [149]. For example, in standard NIST SRM 1950 plasma, GC-EI-MS identified 263 metabolites, versus 93 using PCI and 65 using NCI [150]. In general, EI and CI technologies complement each other: EI provides structural information due to comprehensive fragmentation, and CI provides molecular weight information due to the careful preservation of parent ions.

A high pressure (10–150 mPa) is maintained in the ionization chamber at EI and CI, but ionization can effectively occur at atmospheric pressure as well. Thus, atmospheric pressure chemical ionization (APCI) on model mixtures shows results comparable to EI-MS [151]. Atmospheric pressure photoionization (APPI) also has potential in metabolomics, but primarily for targeted research, such as the analysis of polycyclic aromatic hydrocarbons (PAHs) [103]. Both methods are mild and, noteworthy, can also be used in liquid chromatography, so, if necessary, one mass analyzer can be easily connected to different chromatographic devices.

#### 5.3.2. Mass Analyzers

The mass analyzer follows the ion source in the mass spectrometer design. Its task is to measure molecular ions’ mass-to-charge ratio (*m*/*z*) and their fragments created at the previous stage. The mass analyzer is followed by an electron multiplier, which, using a cascade of conversion dinoplates, multiplies the number of emission electrons and makes it possible to register an output current of several mA as an analytical signal [152].

GC-MS is a mature technology that uses various types of mass analyzers. The sample preparation carried out before the GC-MS analysis makes the metabolomic fraction of plasma and serum suitable to any mass analyzer, for which there are no design problems with pairing to a gas chromatography system. Further, we describe various mass analyzers (quadrupoles, ion traps, Orbitraps, Fourier transform ion cyclotron resonance mass analyzers, and time-of-flight mass analyzers) with references to relevant experimental projects.

Quadrupoles (Q) are the most common analyzers used in GC-MS. The quadrupole mass analyzer consists of four parallel cylindrical metal rods located in a vacuum chamber. An ion moves equidistantly from these electrodes. The magnetic field generated by Q allows only ions with a certain *m*/*z* to pass through. All other ions deviate from a straight path, not reaching the detector. Thus, only one type of ions with a specific *m*/*z* can reach the detector at a time, so the sample is analyzed sequentially by enumerating from the lowest to the highest *m*/*z* ratios. This analyzer is characterized by a wide dynamic range of determined concentrations, high sensitivity but relatively low resolution [12]. In addition, quadrupole mass analyzers have a low scan rate [153,154], which hampers the deconvolution of overlapping peaks. However, the quadrupole allows for reliable and fast metabolic profiling methods. For example, for the analysis of serum, a method was developed that was practically not inferior in quality to the longer methods. Another competitive advantage of such systems is their reliability and relatively low cost [55,56].

Often, such analyzers are stacked in tandems of three series-connected quadrupoles (QQQ), which allows you to create complex strategies for targeted metabolomic profiling. The first and third quadrupoles filter the ions passing through them by mass. The second quadrupole is a collision cell that is filled with gas. The operating mode of each quadrupole can be set separately, adjusting the technical performance under the pressure of the need. The mode assumes the passage of ions through the mass analyzer only with a specific pathogen *m*/*z*. The scanning mode assumes an alternate change in the *m*/*z* ratio, as a result of which it is possible to capture the spectrum of the entire mass range [155]. One of the options for using the QQQ scheme is the multiple reaction monitoring (MRM) of target metabolites [156]. For instance, using ultra-sensitive gas chromatography-tandem mass spectrometry diclofenac without preliminary derivatization was detected and quantified in blood samples, with a linearity range between 0.1–200 ng/mL, the limit of quantification of 0.1 ng/mL, and the limit of detection of 0.05 ng/mL [157].

Another low-resolution mass analyzer used in targeted metabolomics is the ion trap (IT) [158]. The main advantages of such devices are the elegance of design solutions and ease of operation. Using such a mass analyzer in combination with gas chromatography, it was possible to quantitatively measure the content of 17 steroids in blood plasma [159]. The disadvantages of low-resolution systems are primarily related to the difficulties in determining the structure of unknown compounds.

High-resolution detectors include time-of-flight mass analyzers (TOF), Fourier transform ion cyclotron resonance (FT-ICR) instruments, and Orbitraps. FT-ICRs and Orbitraps are also based on trapping ions, but their characteristics differ dramatically from quadrupole mass-analyzers. FT-ICR and Orbitraps provide the unbeaten mass resolution and accuracy of any other mass analyzer, even in routine analyses.

In Orbitrap mass analyzers, *m*/*z* ratios are estimated through the frequency of harmonic oscillations of the ions along the electric field axis. This type of trap provides high sensitivity and resolution (resolution up to 240,000 at *m*/*z* 400 [160]). The enormous capabilities of Orbitrap made it a solution-of-choice in proteomic research [161]; however, these mass analyzers are also widely used in metabolomics [150]. The already mentioned study of standard NIST 1950 plasma demonstrated the advantages of Orbitrap: it separated four times more peaks than the Q mass analyzer (41,588 vs. 8850) [162]. Although the Orbitrap acquisition speed is not fast enough for GC×GC, this mass analyzer performs effectively even in tandem with one-dimensional GC. Thus, in non-human primate serum, the GC×GC-TOF-MS option was able to identify 384 metabolites, while the GC-Orbitrap-MS identified 200 compounds [163].

Ion-cyclotron resonance mass analyzers claim to be the most accurate devices. FT-ICR separates ions by their rotational—cyclotron—frequency in the magnetic field, which is inversely proportional to the *m*/*z*. Fourier transformation is used to get and transform signals from these bouncing and rotating ions [164]. The high accuracy of this method makes it attractive in metabolomic studies [165], although it is achieved at the cost of expensive and bulky laboratory solutions. In general, in metabolomics, FT-ICR is used without preliminary chromatographic separation. However, a successful attempt to combine GC and FT-ICR has been made to analyze small molecules in gasoline samples [166]. FT-ICR coupled with high-performance LC was used to profile the endogenous metabolites in plasma of rats with pyrexia treated with different medicines [167].

The main disadvantage of FT-ICR is the slow speed of spectrum acquisition, up to minutes. The number of points over the chromatographic peak may be insufficient when FT-ICR is combined with modern fast chromatography systems [165]. Several metabolomic studies were performed by DI-MS, direct infusion of the sample into the FT-ICR mass spectrometer [168]. This method is suitable for a qualitative and quantitative metabolic analysis of human plasma. DI-FTICR-MS has demonstrated its effectiveness in rapid metabolic profiling. In a study of blood serum from 49 experimental animals, more than 400 metabolites were detected in about one day of analysis, which is much faster than mass spectrometric analysis with preliminary chromatographic separation [169].

Among high-resolution analyzers in metabolomics, TOF mass analyzers have a reputation for reliable and robust devices. TOF devices analyze how long it takes for ions with different *m*/*z* ratios but the same initial kinetic energy and constant accelerating voltage to cover a fixed distance. The smaller *m*/*z* is, the shorter time will be required to fly through a vacuum chamber.

Time-of-flight analyzers operate with a high scan rate, which allows obtaining sufficient points across the entire peak for better resolution of coeluting peaks. This makes TOF the only mass analyzer fully compatible with GC×GC, which requires a high scan rate [123].

The high resolution of two-dimensional chromatography with a time-of-flight mass analyzer is demonstrated in a study where more than 1000 metabolites were found in blood plasma [134].

Mass analyzers can be combined: so, in addition to the usual sequence of three quadrupole mass analyzers, the various hybrids of mass analyzers are used, e.g., Q-TOF [170] and Q-Orbitrap [171].

Table 2 presents a comparison of the essential characteristics of the most popular mass analyzers used for GC-MS analysis of serum and plasma. Indisputably, for estimation of the performance of a particular mass analyzer, one should consider the analyte and its matrix, the method of preliminary separation, ionization technique, and not only intrinsic characteristics of the device.

The desired “width” of metabolome analysis is also a critical factor in choosing the type of mass analyzers. For example, in panoramic experiments, TOF and Orbitrap analyzers are the most recommendable. In contrast, in targeted approaches, the background signal from a complex matrix is no longer a bottleneck, so triple quadrupoles and ion traps become preferable.

### 5.4. Data Processing

Metabolomics is a data-intensive scientific field. The raw data acquired by tandem chromatography with mass spectrometry are a complex three- (or even four in case of GC×GC) dimensional set of retention times, *m*/*z* values, and their intensities. Interpretation of results obtained in a GC-MS experiment is a delicate process. Each academic group independently chooses to use commercial or freely available software or even create customized scripts.

Commercial software is mature and user-friendly, usually with a developed graphical interface. The vendor supplies such packages along with the equipment, i.a. ChemStation by Agilent Technologies [172], MassLynx by Waters Corporation [173], ChromaTOF by Leco Corporation [174], Compound Discoverer by Thermo Scientific [175], etc. In accordance with text mining results, commercial software accounts for about 38% of computational solutions used for GC-MS data processing [176].

Conversion of the raw data to an open standard format such as mzML allows subsequent processing via vendor-independent software. The most remarkable example of public software is AMDIS [177]. For more than 20 years of its existence, AMDIS has been “enriched” with various extensions improving its operation [178,179]. In toxicological studies of serum samples, automatic evaluation of GC-MS data using AMDIS and its extensions Maurer/Pfleger/Weber identified additional drugs in 17% of samples that had been ignored by experienced personnel during manual data curation [180].

MetaboliteDetector [181], MetaboAnalyst [182], XCMS [183], metaMS [184], MetAlign [185], and MZmine [186] also are successful examples of public software for GC-MS data processing [187]. It is important to note that in data processing, there is a visible trend to shift to online platforms for data analysis, which opens up opportunities for data analysis with only internet access required. Special attention should be paid to the linkage between XCMS and MetaboAnalyst, which allows the researcher to perform a full cycle of metabolomic data analysis: from pre-processing to enrichment analysis, mapping the identified small molecules to metabolic pathways, etc.

**Figure 6:**
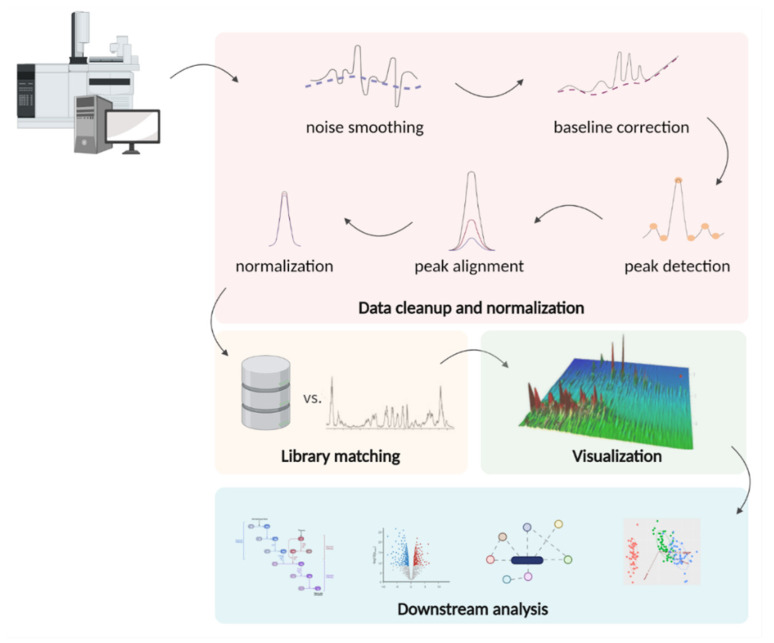
Typical steps in the processing of GC-MS metabolomics data: noise filtering, baseline correction, peak detection, and normalization. The filtered data obtained after preliminary processing is compared with the spectral libraries. The resulting array of annotated spectra can be visualized and used in further statistical algorithms to build biological models.

Both public and commercial packages for GC-MS data processing perform primary preparation of raw files (noise smoothing, baseline correction, feature detection, alignment, normalization), library matching, visualization, and, optionally, downstream analysis (Figure 6).

After carrying out primary processing, allowing to organize the data and check their integrity, one can extract useful information from the cleaned dataset. The chemometric method for extracting such information involves identifying spectral patterns. These patterns can be compared with each other (for example, when analyzing the metabolome of healthy humans and diseased ones), and only then metabolites are identified. However, there are examples of successful barcoding, when characteristic peak patterns can be used to create a digital image of a person without directly identifying compounds [188].

An alternative approach is used in targeted experiments, in which metabolites are first identified, and the result of the identification is then interpreted. In general, the identification pipeline is carried out as follows: preliminary data processing is performed (subtraction of the baseline, marking of signals with an acceptable signal-to-noise ratio, mass spectral deconvolution, alignment, normalization, and calculation of retention indices [189,190] after which the obtained data are compared with the library data (mass spectra, RI).

Once metabolites are identified, downstream analysis can be performed to frame small molecules in terms of current omics knowledge. Multivariate analysis methods are used to extract meaningful information from large sets of experimental data. These methods can be divided into controlled (the data marked by the “supervisor”) and uncontrolled (no “supervisor” required). Principal component analysis, PCA, is the gold standard for interpreting high-dimensional complex datasets. In a multivariate dataset [191], PCA allows identifying class differences in an uncontrolled manner, without information about the class of samples under study. A class can refer to any relevant characteristic, such as diseased patients and healthy subjects [192].

Controlled methods include orthogonal projection to latent structures-discriminant analysis (OPLS-DA), a linear regression method. The original data set is pre-clustered into certain groups, so it is possible to identify the metabolites responsible for their differences. PCA is considered descriptive, and OPLS-DA is deemed to be predictive. In addition to those described above, there are many different chemometric methods of analysis [193].

In addition to standard approaches for statistical modeling, enrichment analysis [194] and network inference [195] methods are used to help integrate metabolomics data into multi-omics models. The development of deep learning (DL) technologies is anticipated in metabolomics. DL is used for peak alignment and annotation, identification, quantification of compounds, integration data with other omics disciplines to build multi-omics models [196,197]. Already, at the primary processing stage of raw data, using trained neural networks, it is possible to filter out up to 90% of false peaks from complex non-target data LC-MS sets without reducing true positive signals. [198]. In matters of building biological models, DL has not yet demonstrated a clear advantage over classical methods of analysis. However, in many studies, this approach showed decent results [196]. For example, comparing DL methods with classical statistical approaches in classification problems on ten clinical metabolic datasets, none of the DLs became the best, although they all showed good or excellent results [199].

There are several challenges in the processing of metabolomics data. On the one hand, the apparent simplicity of the operations performed hides a lack of transparency in data analysis processes because automated pipelines of data processing, in many cases, look like a “black box.” On the other hand, custom in-house solutions often fail to scale from one laboratory to another. Moreover, most of the peaks in the chromatography-mass spectrum remain unidentified even after extensive data processing.

According to some estimates, only 1.8% of the mass spectra are annotated [200], and all other spectra fall into the “dark metabolome” zone [201]. In this light, to increase the number of annotations, bioinformatics algorithms can be used to simulate mass spectra that are absent in libraries based on structural similarity with related compounds already detected by LC-MS/MS [202,203]. On the other side, the transition from direct identification of compounds in the metabolome to barcoding of *m*/*z* features looks especially promising [188].

## 6. Current Challenges and Prospects in Measuring Metabolites

Metabolomics stays at the nexus of chemistry, biology, data science, chemometrics, and bioinformatics. The challenges of metabolomics cannot be solved without crossing scientific boundaries. Therefore, a detailed study of the metabolome requires coordinated teamwork at all stages: from collecting samples to interpreting the obtained data.

Progress in metabolomics (and GC-MS-based metabolomics, in particular) is “fueled” by optimized strategies of sample preparations, numerous technological advances, initiatives on standardization, and collective efforts to generate and curate databases [204,205]. Thus, the Human Metabolome Project [206], which was launched in 2005, has united and streamlined the efforts of the world metabolic community to collect information about the “detectable” human metabolome, mediated by all the other “omics” processes in various states of the organism [207]. Today we are witnessing tremendous growth of HMDB. HMDB 5.0, released in 2021, contains information on more than 220 thousand metabolites, almost twice as much as in 2018 [47]. We believe that the reason for this is the popularization of large-scale omics research “beyond genomics” [208]. The trend of shifting from genome-wide association studies (GWAS) to metabolome-wide association studies (MWAS) has been gaining momentum since 2008. The results of consolidation of several omics layers appear more and more often [209,210,211,212,213].

Metabolomics quickly responds to public challenges: over 200 publications on COVID-19 metabolomics appeared in less than two years [214,215,216]. The spectrum of application of metabolic knowledge solo or as a part of multi-omics is broad: from microbial stains engineering [217] to precise and personalized health monitoring [218,219,220,221].

Unfortunately, clinical metabolic tests are not registered yet [222]. Nevertheless, there are clear signs of the potential of integration of metabolomics into the clinical space [223], which is hampered by insufficiently effective design of data acquisition and (re-)processing [187,224], imperfect standard operating procedures [225], lack of adequate quality controls [69] and unrepresentative samples collections [226].

From a technical point of view, we believe that for comprehensive and rapid metabolomics, the improvements in the system of separation of complex biological mixtures are the most anticipated and promising since the progress of mass spectrometry alone is unlikely to change our understanding of the metabolome qualitatively. As in proteomics, to provide comprehensive coverage of the metabolome requires a shift to multidimensional chromatography techniques, providing state-of-the-art separation [227].

Beyond instrumental challenges, a series of improvements are required to translate metabolomics in routine medical practice, which is not conceivable without extensive population-wide studies explaining how metabolome interacts with phenotype and health status [31].

If these obstacles are overcome, metabolomics tools have tremendous potential to provide solutions for precision medicine and life sciences research. Already today, there are entire platforms of the complete cycle (for example, Metabolon [228]), within the framework of which the design of the experiment, its implementation, and processing of the obtained data are carried out.

Due to their circulating nature, liquid blood components -plasma and serum- are excellent matrices for metabolomic studies [33]. However, the diverse chemistry and wide dynamic range of blood metabolites require digging deeper and developing tailored analytical techniques to provide proper metabolome coverage. We believe that synergy of advanced analytical tools [229], interdisciplinary researches [230], and standardization efforts [225], will increase the rate of integration of blood metabolomics discoveries into practice, providing health professionals, system biologists, data scientists, engineers, and analytical chemists the opportunity to advance their respective industries.

## Figures and Tables

**Table 1:** Major characteristics of NMR, GC-MS, and LC-MS techniques used in metabolomics.

Technique	Major Strengths	Major Limitations	Major Detectable Compounds
GC-MS	Efficient and reproducible chromatography separation Comprehensive mass spectral libraries	Labor-intensive, time-consuming and varying sample preparation procedure Complicated identification of unknown compounds	Volatile and thermo-stable compound Carbohydrates Esters Sterols Steroids Eicosanoids Fatty acids Aminoacids Organic acids Nucleotides and nucleosides Lipids Non-volatile and thermo-labile compounds
LC-MS	Broad range of compounds (including polar, bulky, and thermo-labile metabolites) can be analyzed without derivatization High throughput	Possibility of ion aberrations resulting from a sample matrix Lack of spectral libraries for identification of metabolites	
NMR	Non-destructive analysis High reproducibility Simple or even absent sample preparation	Low sensitivity Relatively high sample volume High cost of apparatus	Carbohydrates Amines Aminoacids and organic acids Bulky molecules

**Table 2:** Comparison of popular mass analyzers used in plasma and serum metabolomics.

Mass Analyzer	Resolution	Mass Range (Da)	Acquisition Speed	Major Benefits	Major Limitations
Quadrupole	~1000	50–6000	Medium	Highly selective Well suited for pairing with GC Relatively cheap Compact	Low resolution Narrow mass range
Ion trap	~1000	50–4000	Medium	Compact Relatively cheap Highly sensitive	Narrow dynamic range Limited resolution Requires pulsed introduction to MS
FT-ICR	over 1,000,000	10–10,000	Slow	High sensitivity High reproducibility High resolving power Wide dynamic range	Expensive and bulky Slow scanning Specific coupling with chromatography systems
Orbitrap	up to 240,000	40–4000	Slow	High resolution Compact and elegant solution	Narrow mass range
TOF	up to 60,000	20–500,000	Fast	Highly sensitive Wide mass range Fast scanning Well suited for pairing with GC	Requires pulsed introduction to MS Requires fast solutions for data acquisition

## Supplementary Materials

The following supporting information can be downloaded at: https://www.mdpi.com/article/10.3390/metabo12010015/s1, Supplementary Material S1, presenting the distribution of physical-chemical properties of blood metabolites deposited in HMDB 5.0.
