# Supplementary material for: Defining Blood Plasma and Serum Metabolome by GC-MS
Source: Metabolites. 2021 Dec 24;12(1):15. doi: 10.3390/metabo12010015 (PMC8779220; doi:10.3390/metabo12010015)

Supplementary material S1

To compare the physicochemical properties, we analyzed blood metabolites (25,408 entries in HMDB 5.0) belonging to the kingdom of organic compounds. The metabolites in HMDB are divided into superclasses - the second level of substance classification according to HMDB Chemical Taxonomy. The metabolites which belong to the same superclass are considered structurally similar. Each superclass curve on the frequency distribution histogram is labeled with a specific color. The monitored parameter is marked on the x-axis, the number of metabolites N (or log $_2$N) with the corresponding properties - along the y-axis.

1. Melting point

The melting point is the temperature at which the substance changes its physical state from solid to liquid. Experimental data presented on the plot was available for 1315 HMDB entries.

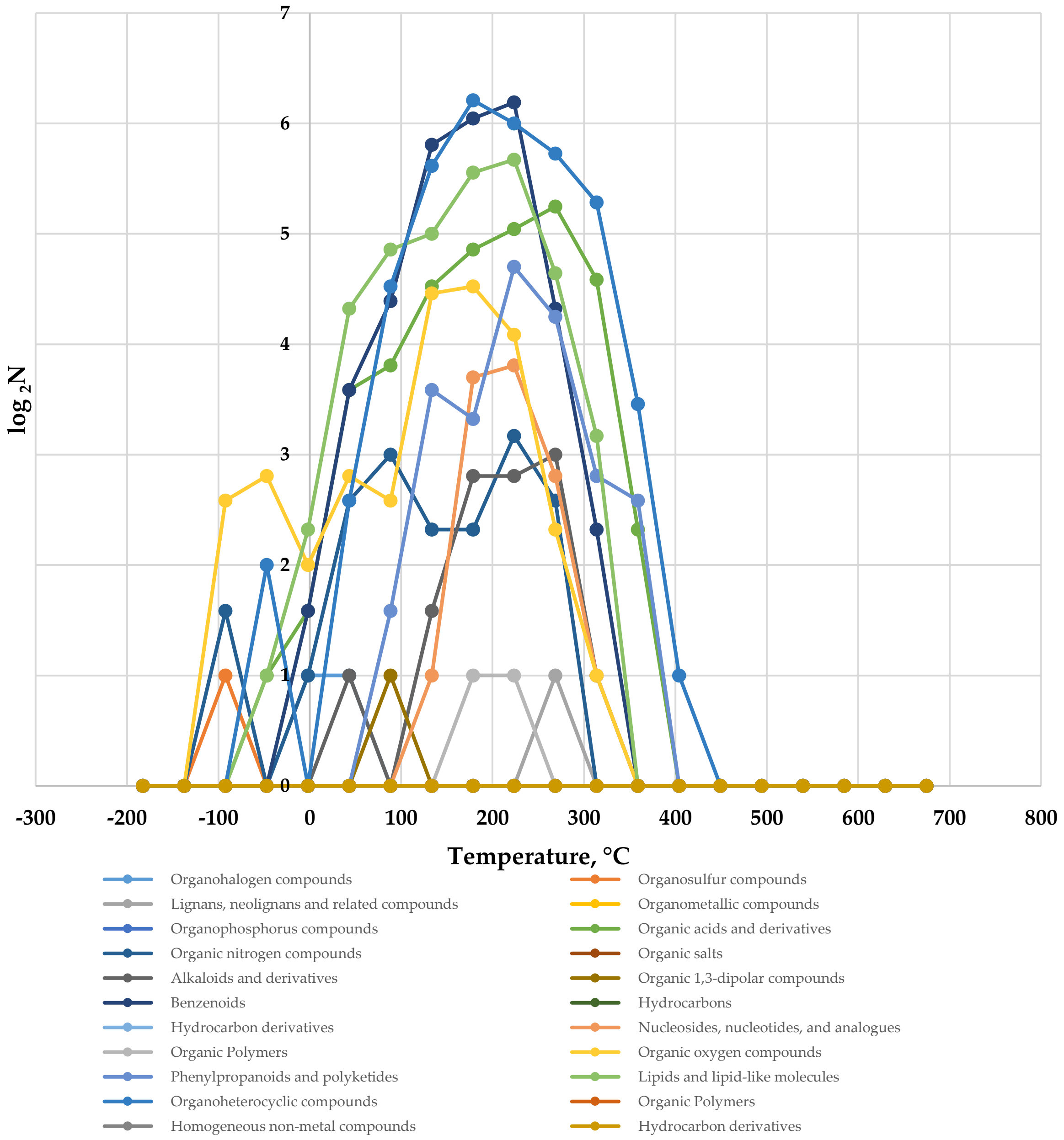

2. Partition coefficient

Partition coefficient (P) is the ratio of concentrations of a compound in a mixture of two immiscible solvents (e.g., octanol and water) at equilibrium. The partition coefficient measures how hydrophilic or hydrophobic a chemical substance is. The partition coefficient is calculated as the ratio of the concentration of the substance in octanol to concentration in water. So, hydrophobic substances have high octanol-water partition coefficients and hydrophilic substances have low octanol-water partition coefficients. To construct the frequency distribution histogram, logP predictions were used, performed for 19,258 metabolites by ChemAxon algorithms.

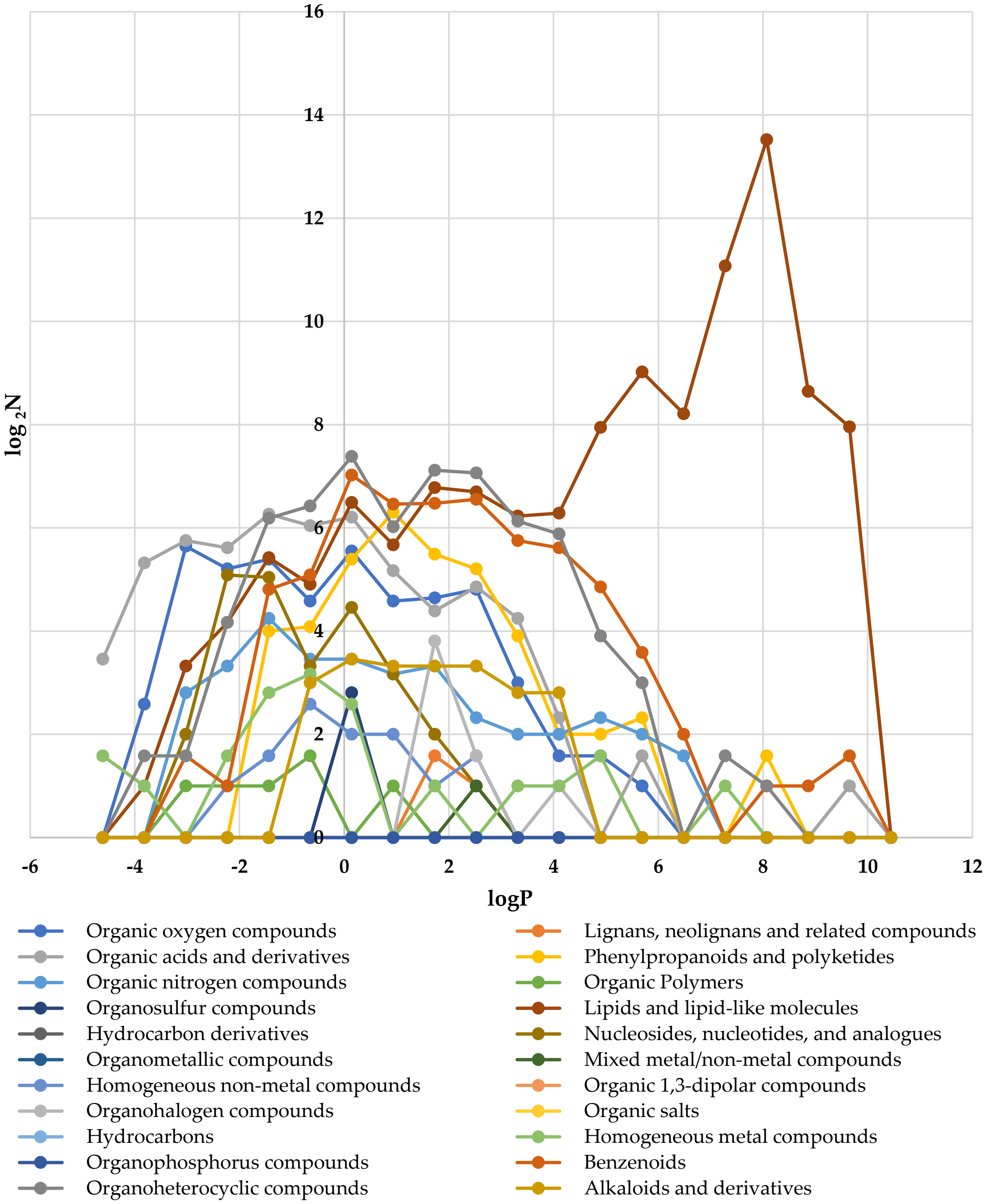

3. **Water solubility**

Water solubility is the ability of a substance to form a solution with water.

- Experimental water solubility was estimated for 1,522 HMDB entries.

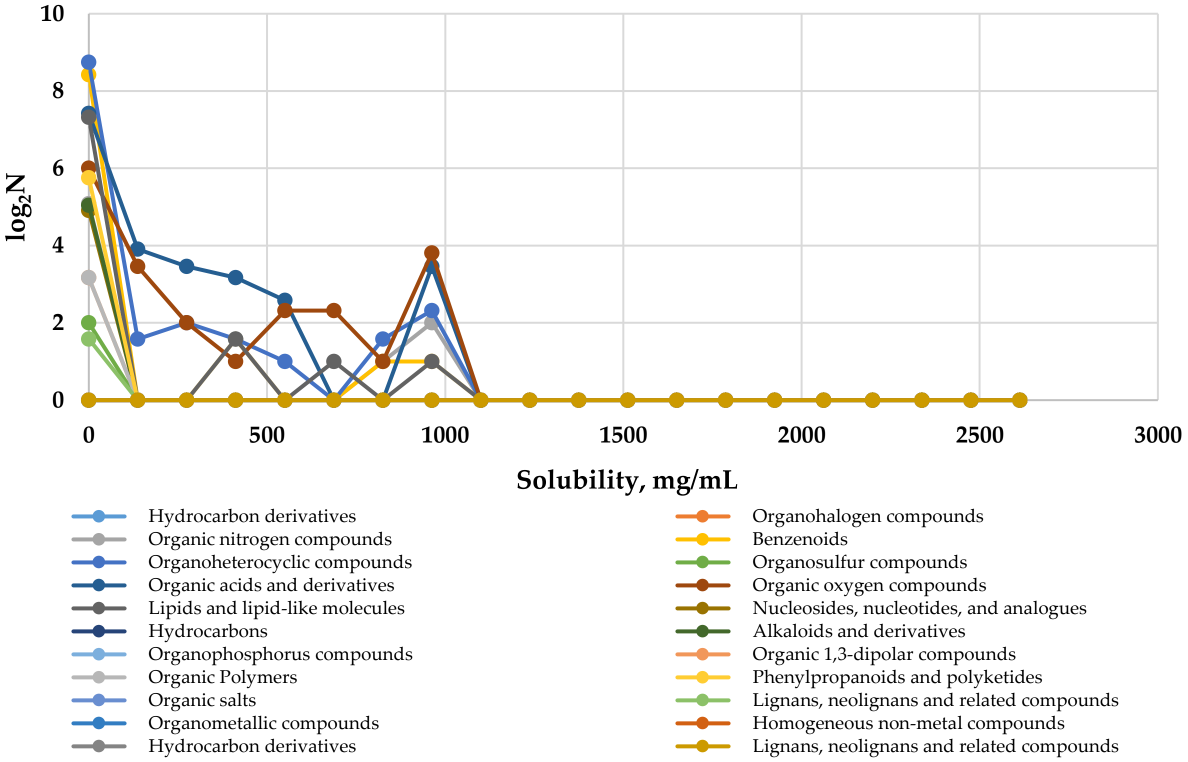

- Theoretical water solubility was predicted for 5,198 HMDB entries by ALOGPS algorithms.

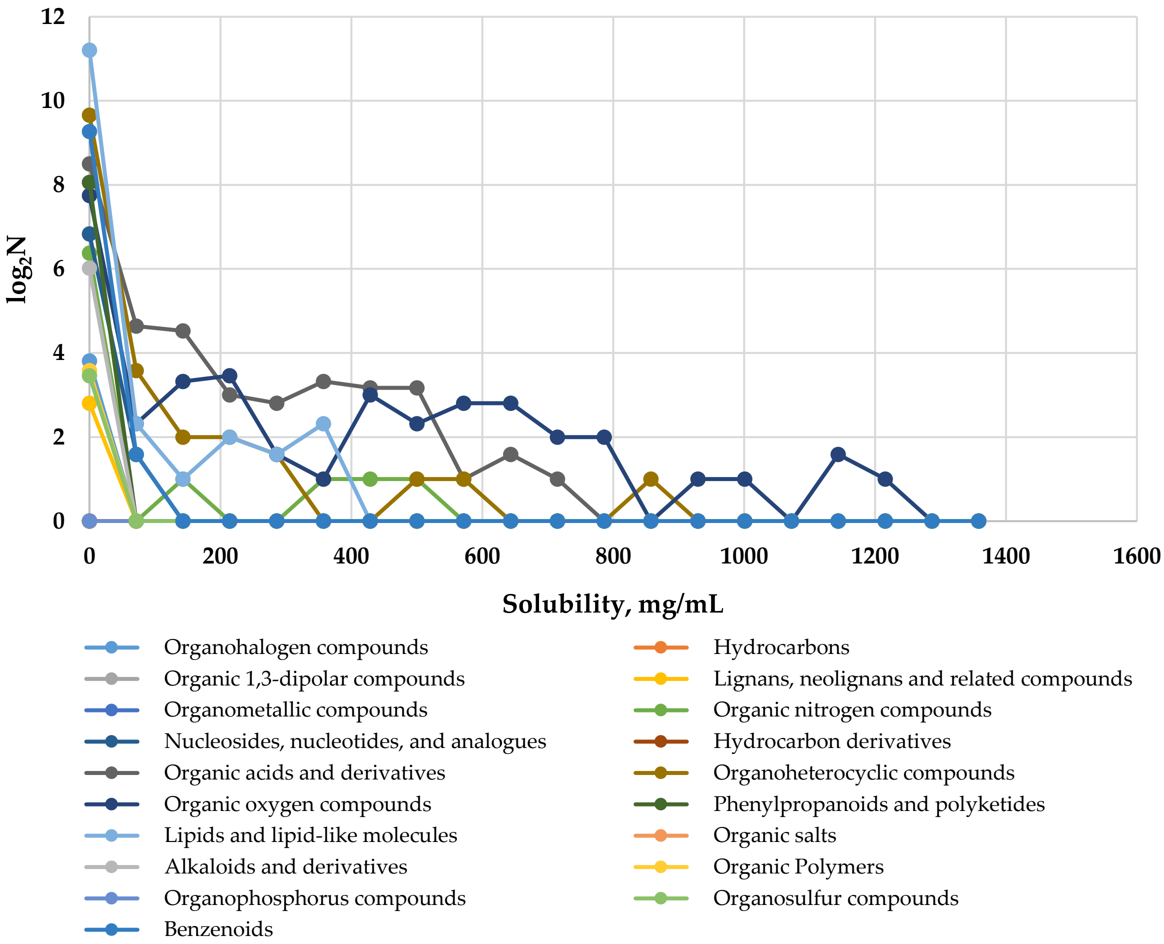

4. **Polar surface area**

Polar surface area (PSA) is the surface sum over all polar atoms or molecules, primarily oxygen and nitrogen, also including their attached hydrogen atoms. PSA theoretical value calculated by ChemAxon is used in practice to estimate polarity. The frequency distribution histogram of PSA was constructed for 19,262 metabolites.

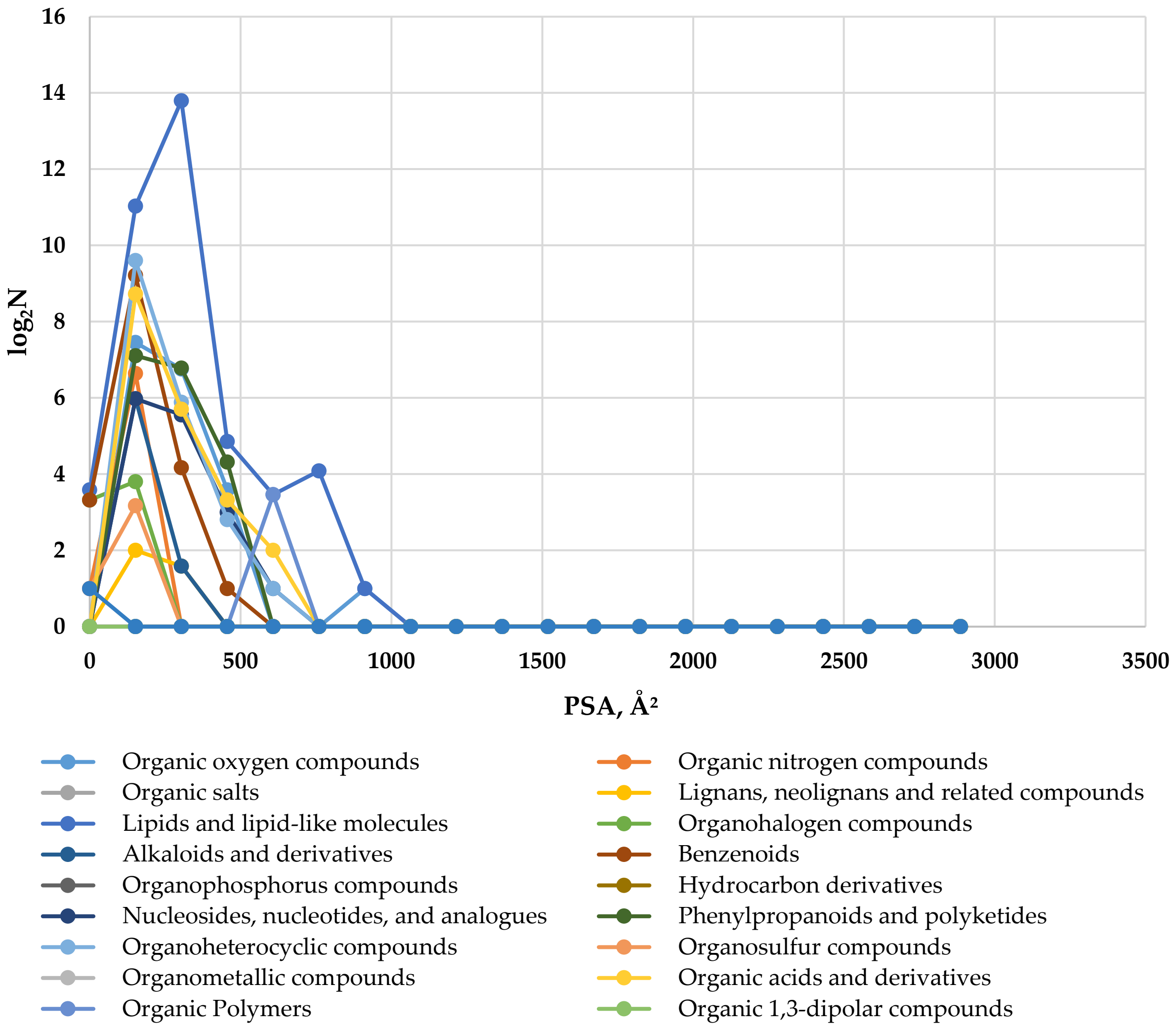

5. **Hydrogen acceptor count**

Hydrogen acceptor count is a theoretical value calculated by ChemAxon for 19,262 metaboltes, predicting the hydrogen bond acceptor properties of the atoms in the molecule. Hydrogen acceptor count is the sum of the acceptor atoms - an atom which always has lone electron pair(s) that is capable of establishing an H bond.

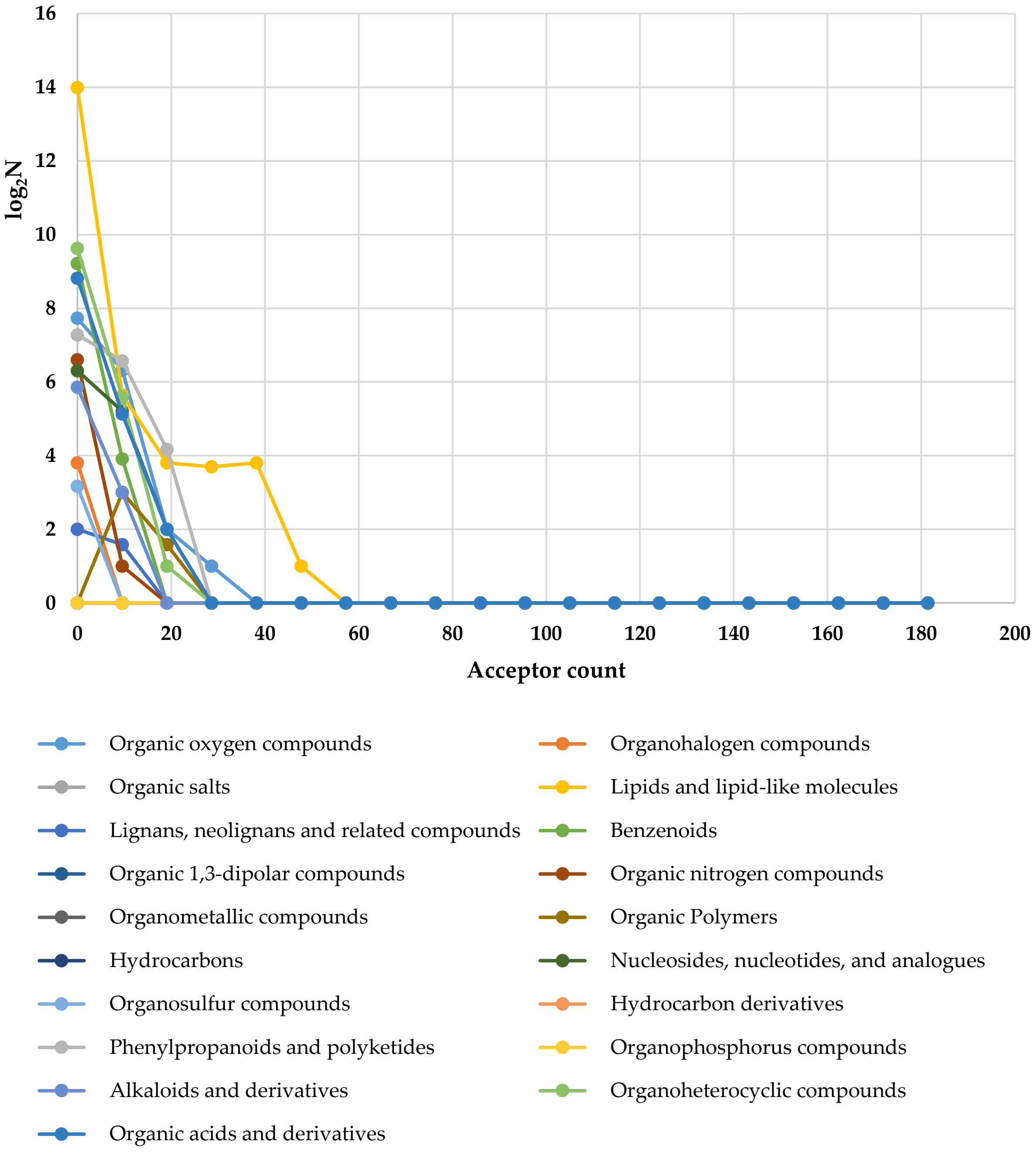

6. Number of rings

Number of rings is a calculated value, performed by the Topological Analysis plugin of the ChemAxon interface for 19,261 metabolites, returning the sum of (hetero)aromatic and (hetero) aliphatic rings.

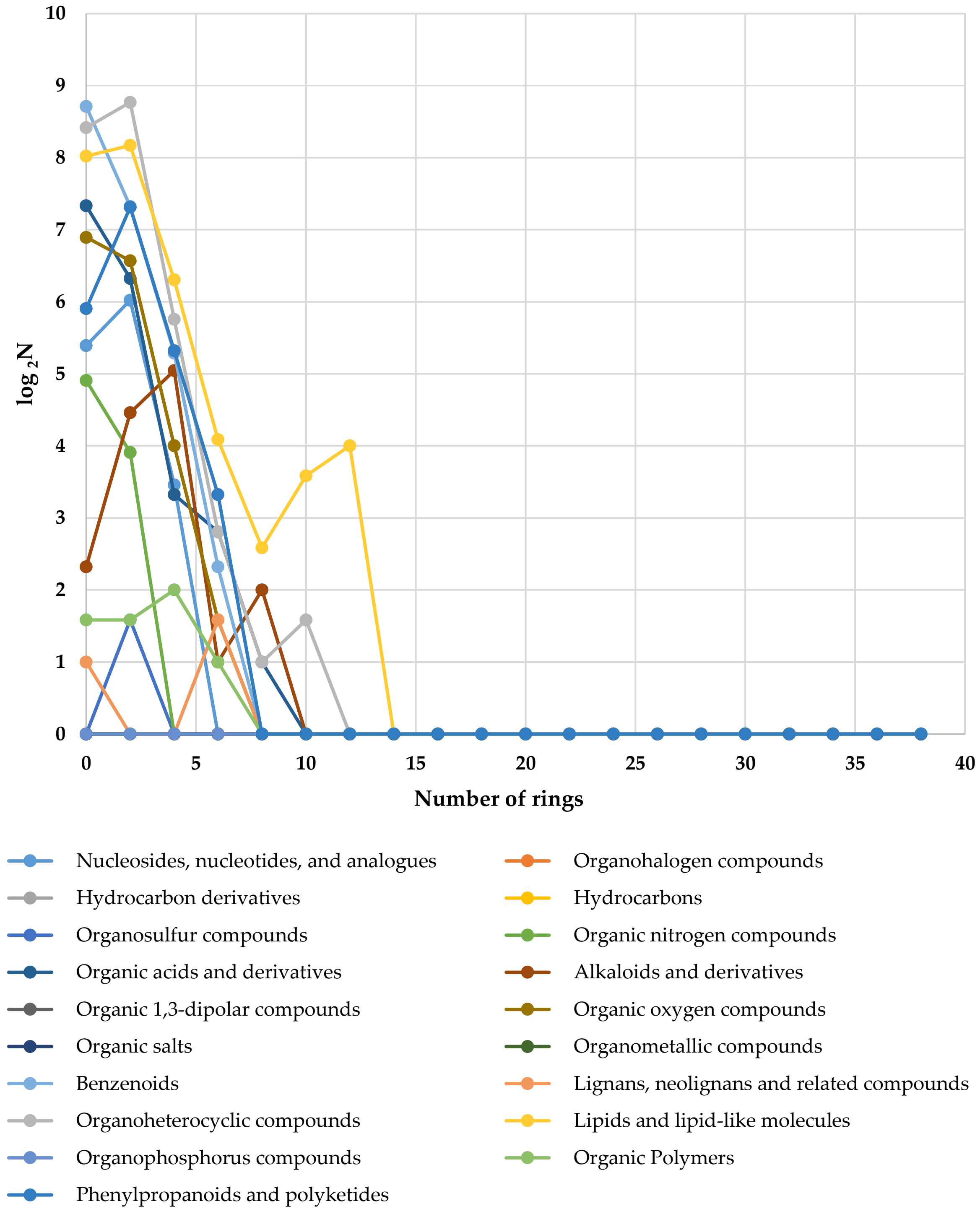

Supplement: Supplementary file 1 [file metabolites-12-00015-s001.zip › metabolites-1502126-supplementary.pdf]
